# The human myocardium harbors a population of naive B-cells with a distinctive gene expression signature conserved across species

**DOI:** 10.3389/fimmu.2022.973211

**Published:** 2022-09-30

**Authors:** Kevin C. Bermea, Nicolas Kostelecky, Sylvie T. Rousseau, Chieh-Yu Lin, Luigi Adamo

**Affiliations:** ^1^Division of Cardiology, Department of Medicine, The Johns Hopkins University School of Medicine, Baltimore, MD, United States; ^2^Department of Pathology, Washington University in St. Louis School of Medicine, St. Louis, MO, United States

**Keywords:** B-cells, heart, cardiology, cardiac immunology, cardiac inflammation, naive B-cells, single-cell sequencing (scRNA-seq)

## Abstract

**Introduction:**

Cardiac immunology studies in murine models have identified a sizeable population of myocardial B-cells and have shown that its modulation represents a promising strategy to develop novel therapies for heart failure. However, scarce data on B-cells in the human heart leaves unclear whether findings in rodents are relevant to human biology.

**Methods:**

We performed immunohistochemical stains to characterize the amount and distribution of B-cells in human hearts, analyzing both fresh and post-mortem tissue. To gain insight into the biology of human myocardial B-cells we analyzed publicly-available spatial transcriptomics and single-cell sequencing datasets of myocardial and peripheral blood mononuclear cells (PBMCs). We validated these findings on primary B-cells sorted from the heart and peripheral blood of left ventricular assistive device recipients. To identify biological pathways upregulated in myocardial B-cells across species, we compared differential gene expression in myocardial vs peripheral blood B-cells across the studied human datasets and published rodent datasets.

**Results:**

In healthy human heart samples, we found B-cells at a ratio of 1:8 compared to T-cells (2.41 ± 0.45 vs 19.36 ± 4.43, p-value <0.001). Myocardial B-cells were more abundant in the interstitium compared with the intravascular space (p-value=0.011), and also more abundant in the myocardium vs. epicardium (p-value=0.048). Single-cell gene expression analysis showed that the human myocardium harbored mostly naive B-cells with a gene expression profile distinct from that of PBMC B-cells. Cross-comparison of differentially-expressed genes in myocardial vs. PBMC B-cells across human and rodent datasets identified 703 genes with consistent differential gene expression across species (binomial p-value=2.9e-48). KEGG pathway analysis highlighted “B-cell receptor signaling pathway,” “Antigen processing and presentation,” and “Cytokine-cytokine receptor interaction” among the top pathways upregulated in cardiac B-cells (FDR <0.001) conserved between species.

**Conclusions:**

Like the murine heart, the human heart harbors naive B-cells that are both intravascular and extravascular. Human myocardial B-cells are fewer and more evenly distributed between these two compartments than rodent myocardial B-cells. However, analysis of single-gene expression data indicates that the biological function of myocardial B-cells is conserved across species.

## Introduction

A growing body of evidence from preclinical models indicates that B lymphocytes have an intimate relationship with the heart. Flow cytometry studies on the digested heart have shown that B-cells are one of the most prevalent immune cells in the mouse heart, at least as prevalent as T-cells (1–4). Parabiosis studies, adoptive transfer studies, and intravital microscopy studies have shown that murine myocardial B-cells are part of a population of recirculating B-cells that transiently adhere to the endothelium (1). Accordingly, the histological analysis found that murine myocardial B-cells are mostly intravascular, with a small contingent of myocardial B-cells found in the interstitium (1, 4). Single-cell transcriptomics analysis has shown that B-cells in the adult murine heart are mostly naive transitional B-cells (1, 4).

Many of the basic aspects of myocardial B-cell biology remain unclear but the available evidence indicates that B-cells play an important role in both myocardial physiology and pathology. In fact, mice with congenital B-cell deficiency were found to have smaller hearts than WT syngeneic controls (1) and B-cells were found to modulate the expression of MHC-II on myocardial resident macrophages (5). In the context of pathology, B-cells were found to be important modulators of myocardial adaptation to injury. B-cell-deficient mice were found to have smaller infarcts and less cardiac dysfunction after permanent coronary ligation (6, 7), small molecule-mediated modulation of B-cell activation was found to improve cardiac function in response to several types of cardiac injury (8), and B-cell depletion was found to improve cardiac function in a model of cardiac hypertrophy/fibrosis (9).

These and other observations have led to the appreciation that B-cells might play an important role in cardiomyopathy and heart failure (10) and might be an important therapeutic target to develop new therapies for these diseases (10–12). However, currently, there is almost no data on B-cells in the human heart. To start addressing this knowledge gap, we used histology and transcriptomics to perform a focused analysis of human myocardial B-cells.

## Methods

### Histologic staining

#### Case selection

Adult autopsy cases for non-ischemic cardiomyopathy (NICM), ischemic cardiomyopathy (ICM), and controls were identified and selected from the archival pathology database at Washington University in St. Louis Department of Anatomic and Molecular Pathology dated between 2018-2020. After reviewing the history and histological findings to confirm the diagnoses, 18 cases of ICM, 7 cases of NICM, and 12 controls were identified. Control cases were selected among decedents with no known cardiac history and for whom the anatomic cause of death was ruled un-related to a cardiac etiology.

Surgical cases for NICM and ICM were identified and selected from the archival pathology database at Washington University in St. Louis Department of Anatomic and Molecular Pathology dated between 2016-2018 for adult left ventricular assist device (LVAD) core material with IRB approval. After reviewing the history and histological findings to confirm the diagnoses, 8 cases of ICM and 15 cases of NICM were identified.

#### Immunohistochemistry of B-cell, T-cell population, and endothelial cells

Immunohistochemistry stains for CD3 (2GV6 clone, Ventana/Roche, Indianapolis, IN), CD20 (L26 clone, Ventana/Roche, Indianapolis, IN), and CD31 (JC70 clone, Ventana/Roche, Indianapolis, IN) were performed on a Ventana BenchMark ULTRA (Ventana/Roche, Indianapolis, IN) per manufacturer protocol in a Clinical Laboratory Improvement Amendments (CLIA) accredited clinical laboratory, with appropriate positive and negative controls. Counts were performed on 10 high-powered fields (10X eyepiece and 40X objective) by evaluating hot-spot locations. All counting was performed by the same pathologist (NK) blinded to the diagnoses. The location of lymphocytes as intravascular vs. extravascular was determined based on morphological features and was confirmed through analysis of adjacent tissue sections stained for CD31 to identify the location of blood vessels.

### Peripheral vs. myocardial B-cells gene expression

#### Data sources

Cardiac single-cell data from the human-Heart Cell Atlas (HCA) project (13) was merged with publicly available data from 20k single-cell peripheral blood cells produced by 10X Genomics (14). For validation of the analysis, we used primary single-cell sequencing data of myocardial and peripheral blood B-cells collected from 2 patients (one with ischemic cardiomyopathy and one with non-ischemic cardiomyopathy) undergoing LVAD implant at Washington University in St Louis, with IRB approval. The left ventricular tissue extracted at the time of LVAD implant was immediately processed via fine mincing and digestion with DNAse (MilliporeSigma), hyaluronidase (MilliporeSigma), and collagenase II (MilliporeSigma). A digestion of 500 mg of tissue was performed in 3 ml of RPMI medium with 120 U of DNAse, 180 U of hyaluronidase, and 1350 U of collagenase. The volume was scaled as necessary to digest all the available tissue. Digestion took place in a shaker incubator, shaking at 300 RPM and 37C for 60’. The digested material was filtered through 40-μm filters and pelleted by centrifugation (250 g for 3 minutes at 4°C). RBCs were lysed by resuspension in an ACK lysis buffer (Invitrogen) for 5 minutes at room temperature. The remaining cells were pelleted, resuspended in 60% RPMI, 20% FBS, 20% DMSO, and frozen at -80°C at a concentration of about 1 gm of tissue per ml of freezing media. For peripheral blood mononuclear cell (PBMCs) isolation, 4 ml of peripheral blood was collected from the patient during the LVAD surgery and processed in parallel with the cardiac tissue samples. PBMCs was isolated using a BD Vacutainer CPT tube. To address the possible confounding effect of tissue digestion on the comparison between myocardial and circulating B-cells, PBMCs were digested with the same enzymes used to digest the myocardium, side by side with myocardial tissue. Immediately after isolation, PBMCs were resuspended in 60% RPMI, 20% FBS, 20% DMSO and frozen at -80°C. After collection and processing of heart and blood samples from 2 patients, digested tissue and PBMCs were thawed on ice. Digested tissue and PBMCs from the two patients were combined. Cells were stained with CD45 (clone 2D1), CD19 (clone H1B19), and propidium iodide for FACS sorting of live CD19+ cells. Digested tissue was also stained with Hashtag antibody 1 (Biolegend, Catalogue #394661) at 1:100, and PBMCs were stained with Hashtag antibody 2 (Biolegend, Catalogue #394663) at 1:100. A total of about 3000 myocardial B-cells and 3000 PBMC-derived B-cells were sorted into RPMI with 1% FCS. Cells were processed with Chromium 5’ Single Cell Kit according to the manufacturer’s instruction and sequenced at a depth of 50K reads per cell. Sample demultiplexing, barcode processing, and single-cell 5′ counting were performed using the Cell Ranger Single-Cell Software Suite (10x Genomics). This dataset is available on the European Nucleotide Archive with accession number PRJEB54024.

To identify the gene expression signature of myocardial B-cells conserved across species we integrated the analysis of previously published single-cell sequencing data from murine myocardial and peripheral blood B-cells (1), human and mouse genes were matched to their orthologous gene using the gene symbol as reference. Approximately 1% of the genes did not show a human orthologous gene.

#### Data analysis

The comparison between the number of B- and T-cells and the comparison between B-cell numbers in various compartments was made using a Poisson test. Observation points that were ±2 standard deviations from the mean were excluded from the statistical analysis. This statistical analysis was performed using standard statistical methods within R software.

For single-cell differential gene expression analysis, a comparison between cell cluster groups of interest was performed in Partek Flow software (version 10.0) using ANOVA. Initially, the data from the HCA and the 10X PBMCs were loaded into Partek Flow, and annotations were added to identify each data source. The HCA data set contains cardiac immune cells previously classified using semi-supervised adversarial neural network for single cell classification (15). Based on the UMAP previously created for the HCA data, B-cells were identified by selecting the clusters with high levels of expression of *MS4A1* (CD20), and T-cells clusters were selected using *CD3G*. Data were normalized by counts per million, using add 1.0 and log 2.0, and features with a value of 0 in 99% of the samples were excluded. The top 20 principal components contributing to data variance were used for principal component analysis. For the generation of UMAP plots, the local neighborhood size was set at 15 with a minimal distance of 0.1, with random initialization. Differentially expressed genes (DEGs) between cell clusters of interest were identified using Gene Set ANOVA using Partek Flow, filtering by the lowest average coverage of 1.0. Pathway enrichment analysis of DEGs was done using DAVID (16). For the 2 validation patients, data were imported into Partek Flow, data were normalized with the settings of add 1.0 and log 2.0. Features where the value was <= 1.0 in at least 90.0% of the cells, were excluded, PCA was made with 20 principal components, and the variance was set as the features contribute. For the UMAP the local neighborhood size was set at 15, a minimal distance of 0.1, distance metric was set as Euclidean, 0 iterations, random generator seed 0, the initialize output values Random, and the number of principal components was set as 20. For validation of gene expression differences between myocardial and peripheral blood B-cells in publicly available datasets and the primary cells analyzed in-house, genes with p-value < 0.05 were selected and a comparison of fold change direction was done using a binomial test (binomial.test() function from R stats package). Pathway analysis was done in the set of genes obtained with fold-change direction match across data sets. Additionally, an Ingenuity Pathway Analysis was done to explore the interactions between the DEGs as well as potential upstream regulators.

*Identification of B-cell subtypes*: B-cell clusters from the HCA were subjected to biomarker profiling (*compute biomarker* function) using Partek Flow with a fold-change threshold of 1.5, the low-filter value was set as lowest average coverage of 1.0. Biomarkers were analyzed using the data browser tool MyGeneset provided by ImmGen (17). With this tool, a comparison of gene expression patterns with a database of each B-cell subtype was made to characterize the nature of the cells. ImmGen ULI RNA-seq and Microarray V1 data sets were matched with the top 20 identified biomarkers.

## Results

### Histologic evaluation

**Figure 1:**
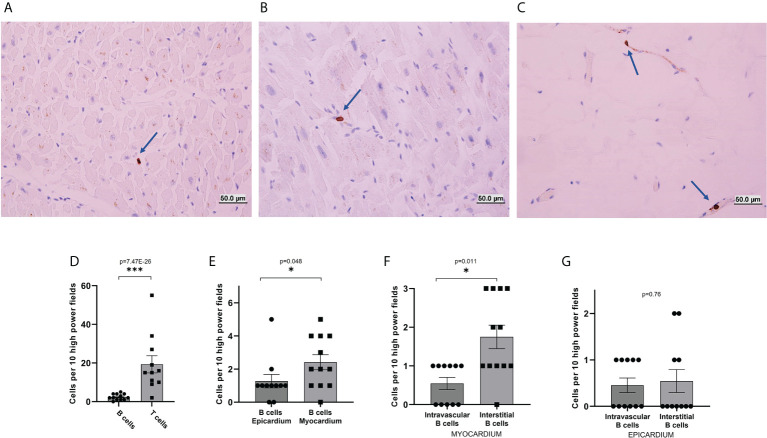
Immunohistochemical analysis of B-cells in human heart sections. **(A–C)** Representative images from CD20 immunohistochemical stains of histological sections of hearts collected post-mortem from decedents without known cardiac pathology. B-cells were found in the interstitial/extravascular space **(A)**, in the intravascular space **(B)**, and the epicardium **(C)**. Blue arrows mark the location of B-cells. **(D–F)** quantification of the number of cells per 10 high power fields. **(D)** The total number of T-cells identified in the myocardial histological sections was about eight times higher than the total number of B-cells (p-value=7.47E-26). **(E)** The total number of B-cells per high power field observed in the epicardium was similar to the total number of cells per high power field observed in the myocardium (p-value=0.048). **(F)** In the myocardium, B-cells were unevenly distributed between interstitial space and intravascular space, with a preference for the interstitium (p-value=0.011). **(G)** In the epicardium, B-cells were evenly distributed between interstitial space and intravascular space, (p-value=0.76). p-values were calculated with a Poisson test. The bars represent average and the error bars represent the SEM. *p-value<0.05; ***p-value<0.001.

Immunohistochemistry for CD3, CD20, and CD31 was performed in 12 post-mortem cardiac biopsies obtained during the autopsy of individuals without any cardiac pathology, to investigate the amount of myocardial CD3+ T-cells and CD20+ B-cells, and to annotate whether B-cells were present in the intravascular or interstitial compartments. We found B-cells in both the extravascular/interstitial space (**Figure 1A**), and the intravascular space (**Figure 1B**). B-cells were present both throughout the myocardium and in the epicardium (**Figure 1C**). T-cells had a similar distribution (**Supplemental Figure 1 A, B**). Endothelial cells were present throughout (**Supplemental Figure 1C**). Staining with the endothelial marker CD31 was used to confirm the location of blood vessels in contiguous sections and thus confirm the intravascular vs. extravascular location of lymphocytes.

We first assessed the total number of B-cells and T-cells per high-power field. We found 2.41 ± 0.45 CD20^+^ B-cells per high-power field. The number of CD3^+^ T-cells was almost 8 times higher (19.36 ± 4.43, p-value=7.47E-26, **Figure 1D**). We then focused on the spatial distribution of B-cells. We first compared the myocardium and epicardium. We found that B-cells were for the most part evenly distributed between the myocardium and epicardium, with a possible slight preference for the myocardium (p-value=0.048, **Figure 1E**). We then assessed B-cell distribution between the interstitial and intravascular space. B-cells appeared to have a prevalence for the interstitial compartment in the myocardium (p-value=0.011, **Figure 1F**) but not in the epicardium (p-value=1, **Figure 1G**).

To rule out the bias introduced from the analysis of post-mortem tissue, we analyzed tissue from 18 autopsy cases of ICM, 7 autopsy cases of NICM, and tissue collected at the time of LVAD placement from 8 cases of ICM and 15 cases of NICM. There was no statistically significant difference in the number and distribution of myocardial B-cells between the autopsy and surgical cases in both NICM and ICM (ICM autopsy vs. surgery in myocardium p-value=0.11 and NICM autopsy vs. surgery in myocardium p-value=1, **Supplemental Figure 2 A, C**). However, statistically significant differences were found in the epicardial distribution of B-cells (ICM autopsy vs. surgery in epicardium p-value=6.82E-08, NICM autopsy vs. surgery in epicardium p-value=4.61E-13, **Supplemental Figures 2 B, D**).

Of note, a small subset of patients (1 NICM and 1 ICM case in autopsy specimens, and 3 NICM and 1 ICM cases in surgical specimens) demonstrated epicardial lymphoid aggregates. These were composed predominantly of T-cells, with a small subset showing a significant B-cell population (**Supplemental Figure 3**). No epicardial lymphoid aggregates were identified in controls.

### Spatial and single-cell gene expression analysis

**Figure 2:**
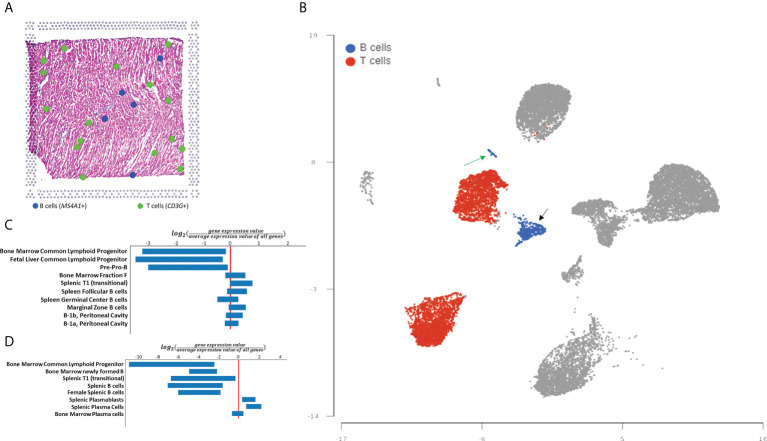
Spatial transcriptomics and single cell-based analysis of human myocardial B-cells. **(A)** Distribution of B-cells, and T-cells in myocardial tissue analyzed with 10X Visium spatial transcriptomics technology. Blue dots represent tissue regions where expression of the B-cell marker *MS4A1* is identified. Green dots represent tissue regions where expression of T-cell gene expression marker *CD3G* is identified. **(B)** UMAP plot of single-cell gene expression analysis of myocardial immune cells from the human Heart CEll Atlas. B-cells are highlighted in blue and T-cells are highlighted in red. B-cells represent 3% of all immune cells (1,196 of 40,868) while T-cells represent 24% of the same population (10,297 of 40,868). **(C)** ImmGen MyGeneSet analysis of the genes with the highest differential expression between the larger B-cell cluster and the rest of the immune cells analyzed. The analysis suggests that this B-cell population is most similar to T1 (transitional) B-cells. **(D)** ImmGen MyGeneSet analysis of the genes with the highest differential expression between the smaller B-cell cluster and the rest of the immune cells analyzed. The analysis suggests that this B-cell population is most similar to plasma cells.

To corroborate and expand on the findings from the histological analysis we turned to genomic analysis. First, we analyzed a spatial transcriptomics dataset of a 10 µm section of fresh frozen human hearts made available by 10X Genomics (13). Analysis of gene expression measured on a section of human myocardium confirmed the presence of B-cells in a lower number than T-cells. In this tissue section, we observed 16 areas with gene expression consistent with the presence of T-cells and 5 areas with gene expression consistent with the presence of B-cells (**Figure 2A**). We then analyzed single-cell sequencing data from the human Heart Cell Atlas (13). Analysis of CD45+ B-cells from the Atlas confirmed that the human heart contains significantly more T-cells than B-cells (24% vs. 3%, **Figure 2B**). Myocardial B-cells were distributed in 2 separate clusters of different sizes (**Figure 2B**). To investigate the biological identity of these two cell clusters we identified the top 20 DEGs in each cluster and compared them to the ImmGen database of gene expression in immune cells (17). **Figure 2C** shows that the larger B-cell cluster showed the highest degree of similarity with T1 naive transitional B-cells, similarity given by the comparison of the gene set used as input in comparison with the mean expression of each gene for all the B-cell selected populations found at the ImmGen database. **Figure 2D** shows that the smaller B-cell cluster showed the highest degree of similarity with plasma cells. **Supplemental Table 1** shows a full list of biomarkers for each B-cell cluster.

**Figure 3:**
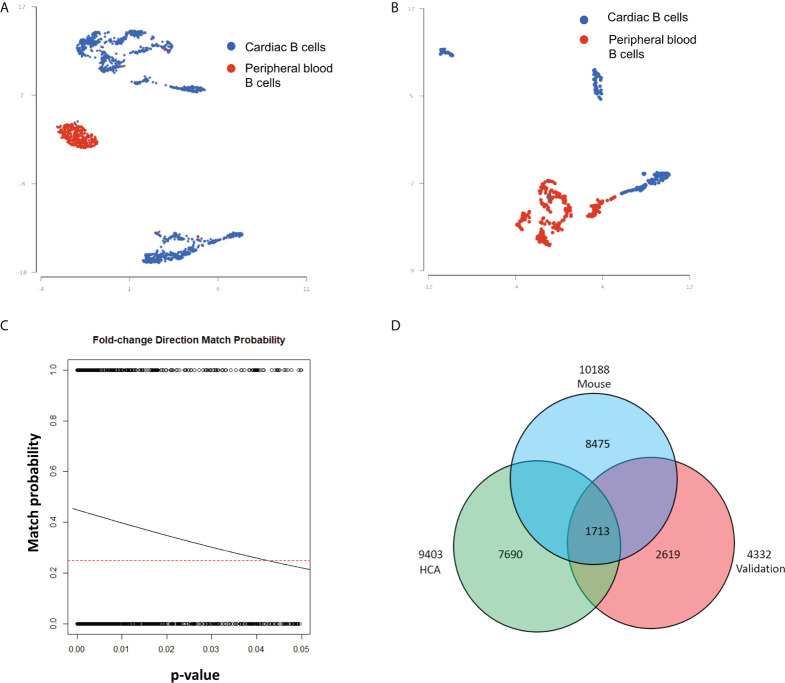
Differential gene expression analysis of myocardial and peripheral blood B-cells reveals characteristics of myocardial B-cells conserved across species. **(A)** UMAP plot of single-cell gene expression of myocardial B-cells from the human Heart Cell Atlas (blue) and 20k peripheral blood mononuclear cells from 10X Genomics (red). Data were merged using Partek Flow. Cells from both sources show important differences in gene expression, however, a small number of peripheral blood B-cells share characteristics with cardiac B-cells and vice versa. **(B)** UMAP plot of single-cell gene expression of myocardial and peripheral blood B-cells collected from the same patients and analyzed within the same gene expression library using Hashtag antibodies. Also, in this “validation” sample myocardial and peripheral blood B-cells have distinct gene expression profiles. **(C)** Logistic regression of the relationship between p-value for a specific gene expression difference vs. the “probability of fold change direction match” observed in differential gene expression analysis of human myocardial vs. peripheral blood B-cells from the publicly available dataset and the dataset collected and analyzed in our laboratory. The dashed line represents the probability of fold change match by random chance (0.25). The lowest the p-value for a gene expression difference between heart and blood B-cells, the higher the chance of concordance between the compared datasets. The solid line represents the observed probability. The greater the incline of the observed probability line, the stronger the match between the two datasets. We observed a very strong match. The probability of obtaining this slope (and therefore this level of matching) between the two datasets just by chance was 2.9 e-59. **(D)** Venn diagram showing the number of DEGs in heart B-cells vs. peripheral blood B-cells in mice (blue), publicly available datasets of human B-cells (green), and the human myocardial and land peripheral blood B-cells analyzed in our laboratory (red). Overlap of 1713 DEGs were seen with 703 following the same fold change direction; binomial test p-value=2.939621e-48.

Studies in murine models indicate that myocardial B-cells have a gene expression signature distinct from that of peripheral blood B-cells. We therefore decided to expand our analysis to assess the gene expression signature of human myocardial B-cells as compared to human peripheral blood B-cells. To this end, we combined B-cells from the human Heart Cell Atlas with B-cells from a publicly available dataset of human peripheral blood (14). **Figure 3A** shows that human myocardial B-cells had a gene expression profile distinct from that of peripheral blood B-cells. Pathway enrichment analysis of DEGs between heart and peripheral blood B-cells revealed “Antigen processing and presentation”, “B-cell receptor signaling pathway”, and “Leukocyte transendothelial migration”, among the top enriched pathways (**Table 1**).

**Table 1:** Pathway analysis of the DEGs between human myocardial B-cells and human peripheral blood B-cells from publicly available datasets.

Pathway	No. of genes in the gene set (K)	No. of genes in overlap (k)	k/K	p-value	FDR
Intestinal immune network for IgA production	49	35	0.71	0	0
Antigen processing and presentation	78	58	0.74	0	0
Retrograde endocannabinoid signaling	148	89	0.6	0	0
Hematopoietic cell lineage	99	67	0.68	0	0
B-cell receptor signaling pathway	82	67	0.82	3.27E-267	3.87E-266
Th17 cell differentiation	108	91	0.84	4.63E-257	5.25E-256
Th1 and Th2 cell differentiation	92	73	0.79	9.08E-254	9.88E-253
Fc gamma R-mediated phagocytosis	97	75	0.77	5.72E-219	5.36E-218
Leukocyte transendothelial migration	114	80	0.7	4.85E-197	4.12E-196
Platelet activation	124	83	0.67	6.33E-167	5.07E-166

In this analysis, we compared two datasets collected at different time points (human Heart Cell Atlas and PBMCs from 10X Genomics). This can introduce bias through several mechanisms, including “batch effect” and the fact that myocardial B-cells were collected through the digestion of tissue while peripheral blood B-cells were not. To validate our findings, we therefore FACS sorted B-cells from human hearts collected at the time of LVAD implant and peripheral blood from the same patients. The peripheral blood mononuclear cells were subjected to the same digestion reaction as the heart tissue and processed in the same single-cell analysis reaction using Hashtag antibodies. **Figure 3B** shows that through this process we observed again that human myocardial B-cells had a distinct gene expression profile from peripheral blood B-cells. The number of cells recovered and analyzed was too small to perform a robust pathway analysis in this sample. However, a comparison of the differential gene expression between myocardial and peripheral blood B-cells between the publicly available datasets and the dataset that we generated showed remarkable concordance. This was assessed through a binomial test of the relationship between concordance in the direction of differential gene expression and the p-value for the gene expression difference (p-value=8.425028e-59, **Figure 3C**). To further investigate the potential bias induced by batch effects, we combined the 4 human datasets (**Supplemental Figure 4A**) and ran the batch effect correction algorithm Harmony (18) (**Supplemental Figure 4B**). **Supplemental Figure 4A** shows that before batch effect correction the myocardial B-cells and peripheral blood B-cells from different sources did not overlap. However, after batch effect correction with Harmony, myocardial B-cells from the human Heart Cell Atlas overlapped with myocardial B-cells from our patients, and peripheral blood B-cells from the publicly available datasets and peripheral blood B-cells from our patients overlapped. Importantly, even after batch effect correction, myocardial and peripheral blood B-cells coalesced in two close but different clusters (**Supplemental Figure 4B**), with a distance qualitatively similar to that, observed in batch effect corrected-comparisons of myocardial and peripheral blood murine B-cells (4).

**Figure 4:**
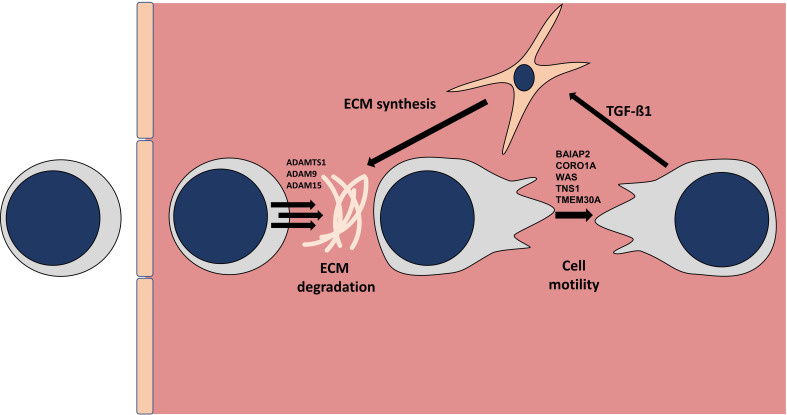
Model of myocardial B-cell behavior/function in the healthy myocardium. Diagram depicting a model of B-cells tissue migration in the myocardial interstitial space. Based on the analysis of the genes specifically upregulated in myocardial B-cells as compared to circulating peripheral blood B-cells we hypothesize that B-cells extravasate, degrade the extracellular matrix (ECM) and crawl through the interstitial space where they communicate with other cells such as fibroblasts. The list of genes involved in matrix degradation and diapedesis was generated using Ingenuity Pathway Analysis.

To get further validation of this observation and gain insight into a possible gene expression signature of myocardial B-cells conserved across species, we performed a Venn diagram-based analysis of DEGs between myocardial and peripheral blood B-cells in 1) publicly available datasets (i.e. human heart atlas B-cells vs 10X genomics PBMCs); 2) in our data set of B-cells collected from the heart and peripheral blood of patients receiving LVAD; and 3) a previously published dataset of murine and peripheral blood B-cells processed in parallel (1). **Figure 3D** shows that 1713 genes were differentially expressed between myocardial and peripheral blood B-cells in all the studied datasets. **Supplemental Figure 5** shows the ranking of these 1713 differentially expressed genes across the 3 datasets. 703 of these genes had matched fold changes across all the 3 datasets recorded (**Supplementary Table 2**). Pathway analysis of the top 200 genes within this group showed that across species myocardial B-cells are characterized by dysregulation of specific immune pathways including “B-cell receptor signaling pathway”, “antigen processing and presentation”, “cytokine-cytokine receptor interaction” (**Table 2**). To further correct for the potential interference of tissue digestion, we performed an additional pathway analysis after removing genes differentially expressed in enzymatically dissociated tissue (19). As shown in **Supplemental Tables 3****,** **4**, the removal of genes modulated by tissue digestion did not significantly modify the findings.

**Table 2:** Pathway analysis of the 200 genes with the lowest p-value among the 703 genes with concordant fold change across murine cells, human Heart Cell Atlas data, and primary sorted cells in myocardial vs. peripheral blood B-cell comparisons.

Pathway	No. of genes in the gene set (K)	No. of genes in overlap (k)	k/K	p-value	FDR
Complement and coagulation cascades	85	8	0.09	1.88E-09	3.44E-07
B-cell receptor signaling pathway	82	8	0.1	3.70E-09	3.44E-07
Hematopoietic cell lineage	99	8	0.08	1.22E-08	7.55E-07
Antigen processing and presentation	78	6	0.08	5.00E-06	1.86E-04
Cytokine-cytokine receptor interaction	295	9	0.03	7.20E-06	2.23E-04
Intestinal immune network for IgA production	49	4	0.08	9.22E-05	1.34E-03
PPAR signaling pathway	75	4	0.05	3.78E-04	4.69E-03
Toll-like receptor signaling pathway	104	4	0.04	1.64E-03	1.69E-02

### Proteases, integrins, and cytoskeleton genes as potential drivers of B-cells patrolling

After a review of the above-mentioned functional pathways, we critically reviewed the list of 703 genes within the identified myocardial B-cells gene expression signature conserved across species. Among these genes are *TGFβ1*, *IL10*, and *MYD88* which are known to participate in cardiac disease. In addition, myocardial B-cells are characterized by increased expression of *APOE*, which is involved in lipid presentation (20). Moreover, the transcripts for the proteases *ADAMTS1*, *ADAM9*, and *ADAM15* were markedly upregulated in myocardial B-cells. Moreover, the integrins *ITGA4*, *ITGA6*, *ITGA9*, and *ITGAV* were expressed by cardiac B-cells together with other genes such as *BAIAP2*, and *CORO1A* that are involved in cytoskeleton reorganization and cell motility (21, 22). Based on this, we hypothesize that in response to cytokine-mediated signaling, cardiac B-cells extravasate into the myocardial interstitium to perform immune patrolling functions. We hypothesize that they “open their way” using proteases, reorganize their cytoskeleton to move through the tissue, and use integrins to connect with the surrounding extracellular matrix until an external signal triggers B-cell receptor-mediated signaling and/or antigen presentation (**Figure 4**). The full list of the 703 genes that showed matched fold change direction across the 3 data sets can be found in the **Supplemental Table 4**.

## Discussion

We report the first systematic analysis of human myocardial B-cells. Using histology, spatial transcriptomics, and single-cell analysis data, we found that the human myocardium harbors a small population of B-cells that is significantly smaller than the pool of myocardial T-cells (**Figure 1**). Through the analysis of histological sections, we found that myocardial-associated B-cells are mostly located in the interstitial space (**Figure 1**). Using single-cell sequencing we found that human myocardial B-cells are naive transitional B-cells, with a small component of plasma cells (**Figure 2**). Comparing the single-cell gene expression signature of human and murine myocardial B-cells we identified a gene expression signature of myocardial B-cells conserved across species (**Figure 3**, **Table 2**). These findings broaden current knowledge of B-cells in peripheral, non-lymphoid tissues (23), inform research on myocardium-B-cell interactions in preclinical models, and contribute to a growing body of knowledge that suggests that the current model of B-cell recirculation might need revision.

We found that the prevalence and distribution of myocardial-associated B-cells in the human heart are markedly different than what has been previously described in the murine heart. Murine studies have identified B-cells as one of the most prevalent myocardial immune cells, with a representation comparable if not superior to that of T-cells (1, 8). Here, we report that the human heart has significantly fewer B-cells than T-cells. The ratio between T-cells and B-cells was about 8:1 in the histological analysis (**Figure 1D**), 3:1 in the spatial transcriptomics analysis (**Figure 2A**), and 8:1 in the single-cell analysis (**Figure 2 B**). The difference in the ratio observed is likely the result of differences in the 3 experimental techniques. The data collected through histology is likely the most accurate. Single-cell sequencing data can be biased by the inefficiency of tissue digestion, and spatial transcriptomics analysis is limited to the analysis of a single tissue section and by “supra cellular” resolution. Histology can be biased by the source of the tissue analyzed, fresh vs. post-mortem, but we verified that the source of tissue did not introduce significant bias in our analysis of the myocardium (**Supplemental Figure 2**). We found a difference between post-mortem and fresh tissue in epicardial B cell prevalence. We speculate that this difference might result from the fact that all fresh tissue analyzed were left ventricular cores, which therefore included epicardial tissue from the left ventricular apex, while the anatomical origin of the epicardial post-mortem tissue varied. Importantly, all techniques we used pointed to a significantly higher prevalence of T-cells than B-cells.

In terms of distribution, studies in murine models found that myocardial B-cells are mostly intravascular, in close association with the endothelium (1, 4), with a ratio of intravascular vs. interstitial B-cells of 10-20:1 (1). We found that instead human myocardial B-cells have a preference for the interstitial space. We analyzed the intravascular vs. interstitial location of B-cells by integrating the assessment of a trained pathologist on slides stained for a B-cell marker with nuclear counterstain with staining for the endothelial marker CD31 in adjacent sections. This method is not as accurate as co-staining in the same slide, and therefore a small percentage of B-cells might have been inaccurately assigned to either of the two spaces. However, our findings clearly show that the distribution of B-cells between the intravascular and interstitial space in the human myocardium is different than in mice (1, 24). We speculate that the different number and distribution of B-cells between rodents and humans might reflect the marked difference in the number of circulating B-cells between the two species. While in humans B-cells account for approximately 5-10% of lymphocytes in peripheral blood (25, 26) and they are only approximately 150-300 per microliter (25), in mice, B-cells account for close to 50% of lymphocytes in peripheral blood and they are approximately 2-4,000 per microliter (27).

We found two important similarities between murine and human myocardial B-cells in terms of identity and gene expression signature. First, we found that human myocardial-associated B-cells are naive transitional B-cells. This is in line with what has been previously described for murine myocardial B-cells (1). Second, differential gene expression analysis of human myocardial associated B-cells and peripheral blood B-cells highlighted that myocardial B-cells have a distinct gene expression signature that is enriched in specific immune pathways such as “B-cell receptor signaling”, “antigen processing and presentation”, and “leukocyte transendothelial migration”. These findings are remarkably similar to those obtained through differential gene expression analysis of murine myocardial and peripheral blood B-cells (1), with 4 of the top 10 enriched pathways being the same. We corroborated this finding, produced through the analysis of publicly available datasets, with the analysis of human myocardial and peripheral blood B-cells collected from two patients. The analysis of publicly available datasets is limited by the potential interference of “batch effects” and our analysis of primary B-cells is limited by the fact that we were able to successfully sequence only a very small number of cells. However, the remarkable level of concordance (p-value < 0.0001) in the binomial test of fold change direction between the myocardial vs. peripheral blood gene expression analysis of the two datasets provided the corroboration we were seeking.

The observation that DEGs between myocardial and peripheral blood B-cells are similar in humans and mice is probably the most important observation in this study, as it suggests that the biological function of myocardial B-cells might be conserved across species. To strengthen this observation, we performed a Venn diagram-based analysis to identify a gene expression signature of myocardial B-cells conserved across species. In this analysis, we used the differential gene expression analysis of myocardial vs. peripheral blood B-cells from publicly available human datasets and the differential gene expression analysis from myocardial and peripheral blood B-cells analyzed in our laboratory as independent datasets. Since the primary dataset we generated has a small number of cells, this choice reduced our statistical power but, at the same time, biased us toward highlighting the strongest biological signature of myocardial B-cells. Remarkably, this analysis highlighted again the “B-cell receptor signaling” pathway and the “antigen processing and presentation pathway”, which were identified as a hallmark of myocardial B-cells in the first studies in murine models (**Table 2**).

Seminal studies from Gowan and collaborators (28, 29) from the 1960s informed the current model of B lymphocyte recirculation which states that B lymphocytes continuously recirculate between primary and secondary lymphoid organs through blood and lymph (30). This model has been challenged by reports of naive B-cells in the murine lung (4), heart (1), and other tissues (23). Naive B-cells in the peripheral tissue were shown to be, at least for the most part, in the intravascular space (1, 4, 5, 31). This suggested that the “dogma of B-cell recirculation” might need some revision because naive B-cells can adhere to the endothelium and pause during their recirculation. Adhesion of naive circulating B-cells to the endothelium was indeed observed with intravital microscopy in the heart (1), lung (4), and kidney (32). Our transcriptomics analysis further supports the need for this revision of the current model of B-cell recirculation, but it goes a step further suggesting that naive B-cells might also exit the vasculature and enter the uninjured heart tissue. Further targeted studies will be needed to confirm our observation. However, based on our analysis we propose that a sub-population of naive circulating B-cells might exit the circulation to perform immune patrolling in tissues (**Figure 4**).

Our focused analysis of B-cells from the human Heart Cell Atlas highlighted the presence of a small subset of plasma cells in the heart. Plasma cells can migrate to chronically inflamed tissue (33) and they have been previously described in transplanted hearts with allograft vasculopathy (34). However, we are not aware of prior studies reporting the presence of plasma cells in healthy hearts. The number of plasma cells identified is too small to draw any conclusions. However, it raises the intriguing possibility that the heart might harbor a previously unappreciated rare population of tissue plasma cells.

Our histological analysis highlighted occasional B- and T-cell clusters in the epicardium of patients with cardiomyopathy. Endocardial lymphoid aggregates with admixed B- and T-cells are frequently encountered in transplanted hearts and are typically considered a benign finding (35, 36). To the best of our knowledge, large lymphoid aggregates have not been described before in the epicardium of native hearts with cardiomyopathy. We speculate that these lymphoid aggregates might constitute tertiary lymphoid organs that are produced in response to chronic myocardial inflammation. However, further studies focused on the epicardium will be needed to understand the potential biological significance of this observation.

## Conclusion

In conclusion, through the first focused analysis of human myocardial B-cells, we found that the human heart hosts a small population of naive B-cells that are divided between the intravascular space and the interstitial space, with a slight preference for the interstitium. While this is significantly different from what has been observed in murine models, we found that myocardial B-cells in both humans and rodents are characterized by a dysregulation of pathways related to “B-cell receptor signaling”, “antigen processing and presentation”, and “cytokine-cytokine interactions”. This suggests that myocardial B-cells might have a conserved function across species and that findings on B-cells-myocardial interactions from pre-clinical models might be relevant to humans. Further studies will be needed to characterize the functional role of B-cells in the human heart.

## Data availability statement

The data presented in this study are deposited in the European Nucleotide Archive, accession number PRJEB54024.

## Ethics statement

The studies involving human participants were reviewed and approved by Institutional Review Board of Washington University in St Louis. The patients/participants provided their written informed consent to participate in this study.

## Author contributions

LA and C-YL designed the study. NK performed the analysis of histological sections. LA performed the single-cell analysis of primary human B-cells. LA and KB analyzed data and wrote the first draft of the manuscript. SR assisted with data analysis. All authors contributed to the writing and reviewed and approved the manuscript.

## Funding

This study was funded by an internal grant from the Washington University in St Louis Institute for Clinical and Translational Sciences awarded to Luigi Adamo and by NHLBI grants 5K08HLO145108-03 and 1R01HL160716-01.

## Conflict of interest

Luigi Adamo is co-founder of i-Cordis, LLC, a start-up company focused on the development of immunomodulatory molecules for the treatment of heart failure, with a specific interest in derivatives of the B-cell modulating drug pirfenidone. The remaining authors declare that the research was conducted in the absence of any commercial or financial relationships that could be construed as a potential conflict of interest.

## Publisher’s note

All claims expressed in this article are solely those of the authors and do not necessarily represent those of their affiliated organizations, or those of the publisher, the editors and the reviewers. Any product that may be evaluated in this article, or claim that may be made by its manufacturer, is not guaranteed or endorsed by the publisher.
