# Supplementary material for: The human myocardium harbors a population of naive B-cells with a distinctive gene expression signature conserved across species
Source: Front Immunol. 2022 Sep 30;13:973211. doi: 10.3389/fimmu.2022.973211 (PMC9563334; doi:10.3389/fimmu.2022.973211)
Supplement: Supplementary file 1 [file DataSheet_1.docx]

*Supplementary material*

**Supplemental figures**


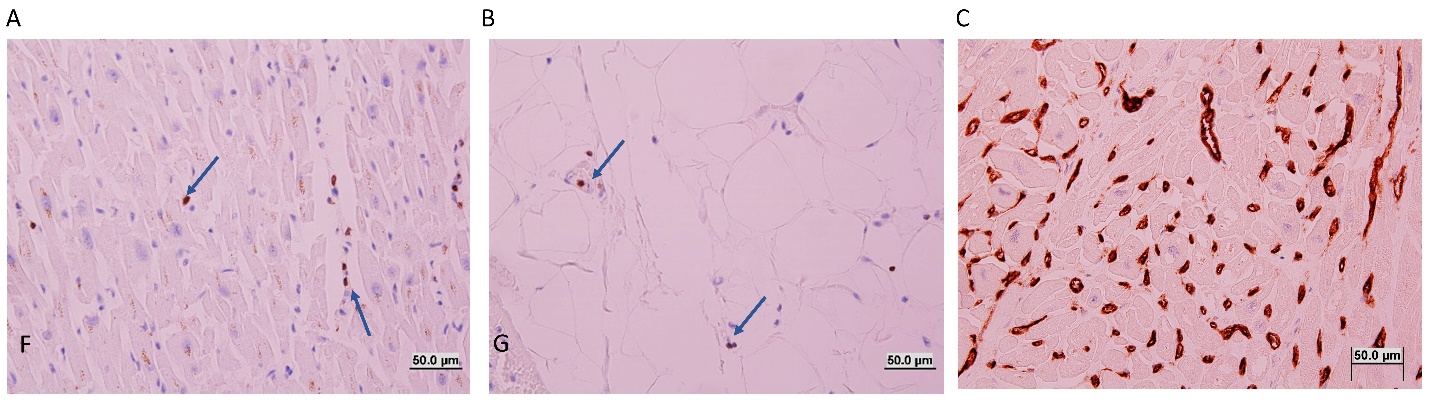


**Supplemental Figure 1. Representative images of immunostaining for B cells and endothelial cells.** A) representative staining of CD3+ T cells in the myocardium. B) representative staining of CD3+ T cells in the epicardium. C) representative staining of CD31+ endothelial cells.


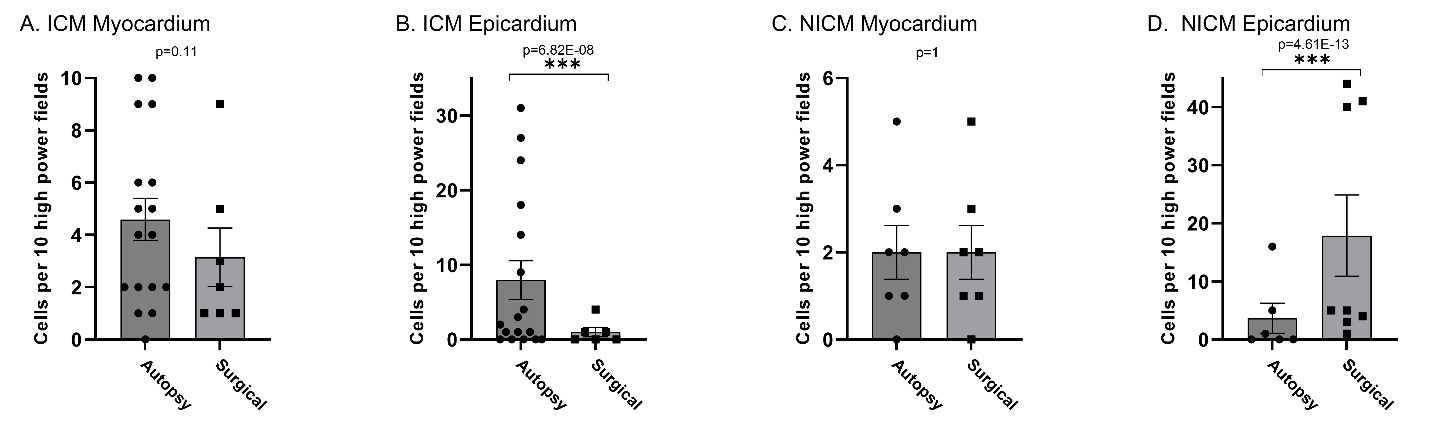


**Supplemental Figure 2. Analysis of B cells in fresh vs post-mortem myocardial tissue.** Bar chart of the number of cells quantified in hearts collected from banked autopsy specimens and surgical specimens. The analysis shows autoptic heart tissue from patients with ICM or NICM and VAD cores (surgical samples) from patients with ICM and NICM. A) Comparison of the abundance of B cells in the myocardium in post-mortem and surgical samples obtained from patients with ICM. B) Comparison of the abundance of B cells in the epicardium in post-mortem and surgical samples obtained from patients with ICM.C) Comparison of the abundance of B cells in the myocardium in post-mortem and surgical samples obtained from patients with NICM. D). Comparison of the abundance of B cells in the epicardium in post-mortem and surgical samples obtained from patients with NICM. The number of myocardial B cells observed in both the epicardial region and the myocardium was similar in post-mortem and surgical tissues for both ICM and NICM. The bars represent the average and the error bars represent the SEM. All p-values were calculated using the Poisson test. *** p<0.001; ** p<0.01; * p<0.05.


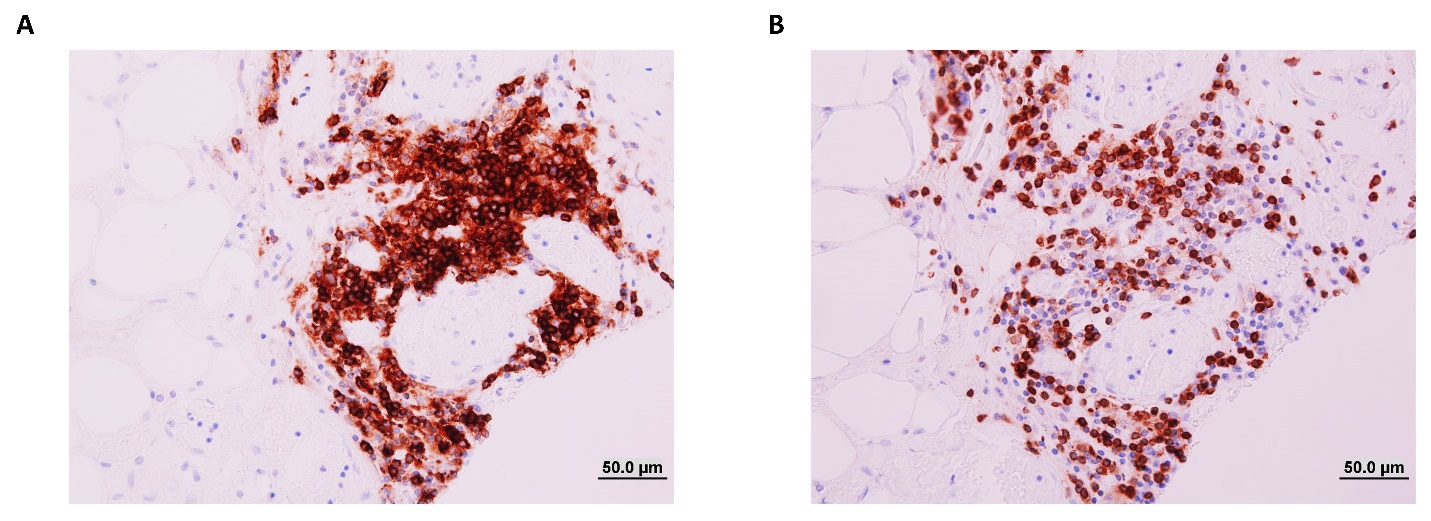


**Supplemental Figure 3. Representative epicardial lymphoid aggregates.** Immunohistochemistry staining for B and T cells in epicardial tissue. – A) The image shows B cells (CD20+) from a lymphoid aggregate. B) The image shows T cells (CD3+) from the same lymphoid aggregate.


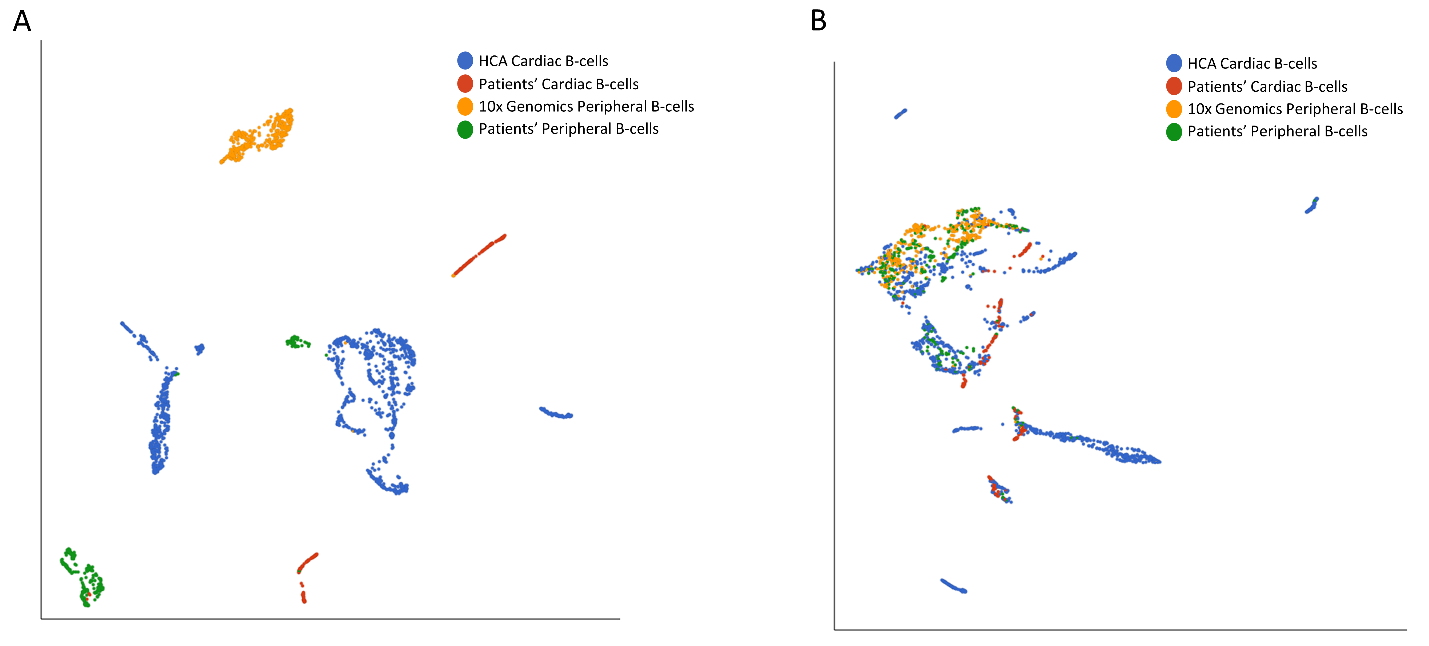


**Supplemental Figure 4. Integration of the 4 human B-cell data sets before and after batch correction.** A) UMAP plot showing the distribution of the four data sets before performing batch correction with Harmony. B). UMAP plot showing the distribution of the four data sets after performing batch correction. An overlap of the PBMCs from the 10x genomics publicly available data set and the PBMCs derived from the two patients included in this study can be visualized.


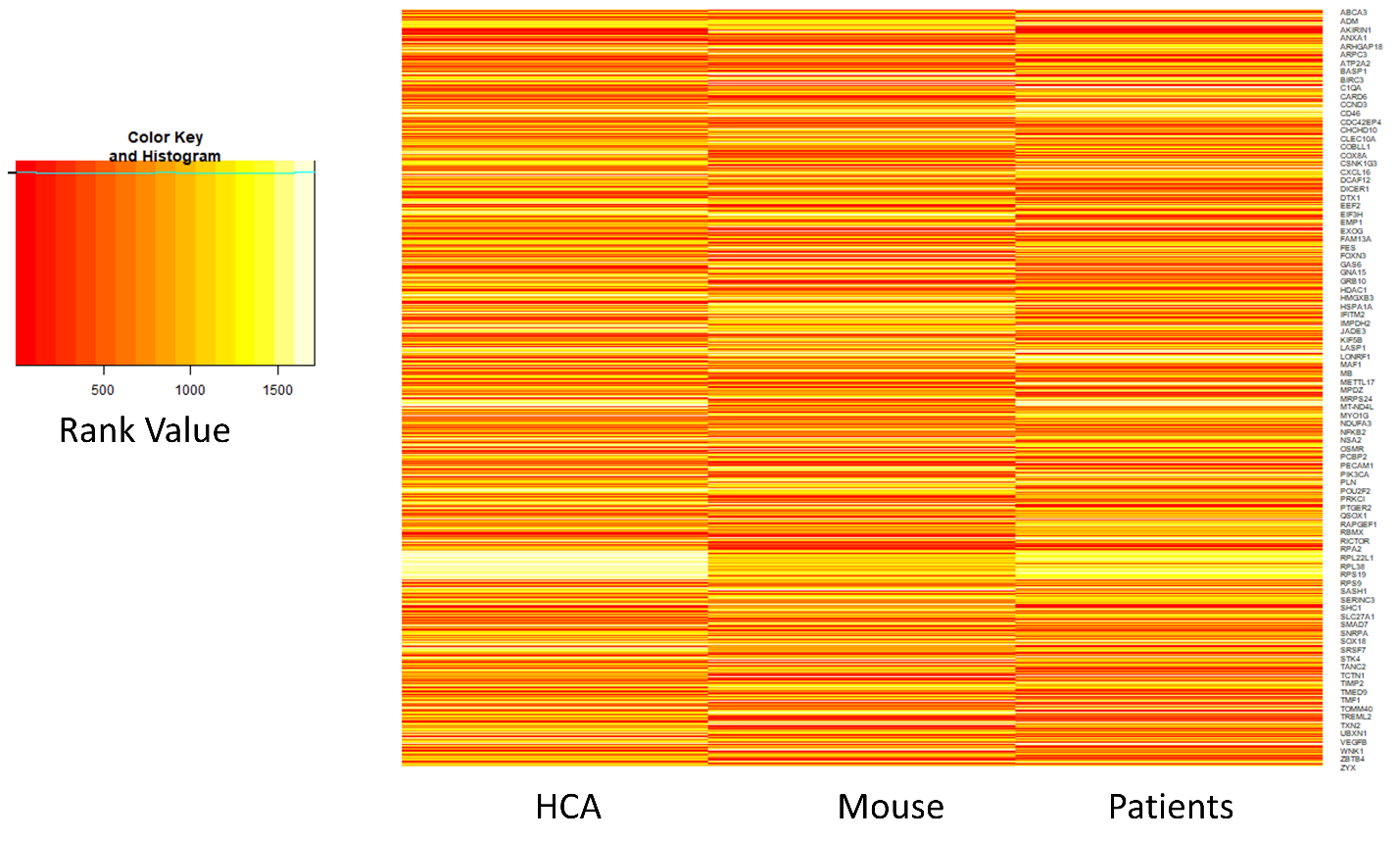


**Supplemental Figure 5. Heatmap of the ranking of the heart vs blood B-cell differentially expressed genes of the three data sets.** Genes were ranked from 1 (red) to 1713 (yellow) according to their p-values to assign color and were listed in alphabetical order from top to bottom to generate the heatmap plot.
